# Thrombotic Thrombocytopenic Purpura: A Rare Cause of Severe Acute Kidney Injury

**DOI:** 10.7759/cureus.24221

**Published:** 2022-04-17

**Authors:** Hatem Najar, Laurene Tuider, Vinita Kukkar, Mohammad Quasem

**Affiliations:** 1 Internal Medicine Department, United Health Services Hospitals, Johnson City, USA; 2 Pathology Department, United Health Services Hospitals, Johnson City, USA; 3 Nephrology Department, United Health Services Hospitals, Johnson City, USA

**Keywords:** acute kidney injury, therapeutic plasmapheresis, thrombocytopenia, thrombotic thrombocytopenic purpura, thrombotic microangiopathy (tma)

## Abstract

Thrombotic microangiopathy (TMA) is a serious and potentially fatal disorder, especially if there is a delay in diagnosis and appropriate treatment. Thrombotic thrombocytopenic purpura (TTP) and hemolytic uremic syndrome (HUS) are the two main forms of TMA. Although severe acute kidney injury (AKI) is a common manifestation of TMA, it remains rarely described in reported TTP cases. We present a rare case of TTP in a 76-year-old African American male who presented with severe AKI (stage 3) and uremic symptoms. Early diagnosis and prompt treatment of TTP with plasmapheresis followed by rituximab and caplacizumab were associated with the resolution of the AKI and avoidance of hemodialysis. This case highlights the need to consider TTP as a possible diagnosis even in the setting of severe AKI.

## Introduction

Thrombotic microangiopathy (TMA) is a serious and fulminant disorder characterized by microangiopathic hemolytic anemia, thrombocytopenia, and various organ damage [1,2]. Thrombotic thrombocytopenic purpura (TTP) is one of the major types of TMA. TTP is more characterized by severe thrombocytopenia and neurological symptoms. Although severe acute kidney injury (AKI) is a common manifestation of TMA, it remains rarely described in reported TTP cases [3]. We present a rare case of TTP in which the patient presented with severe AKI and uremic symptoms.

## Case presentation

**Figure 1:**
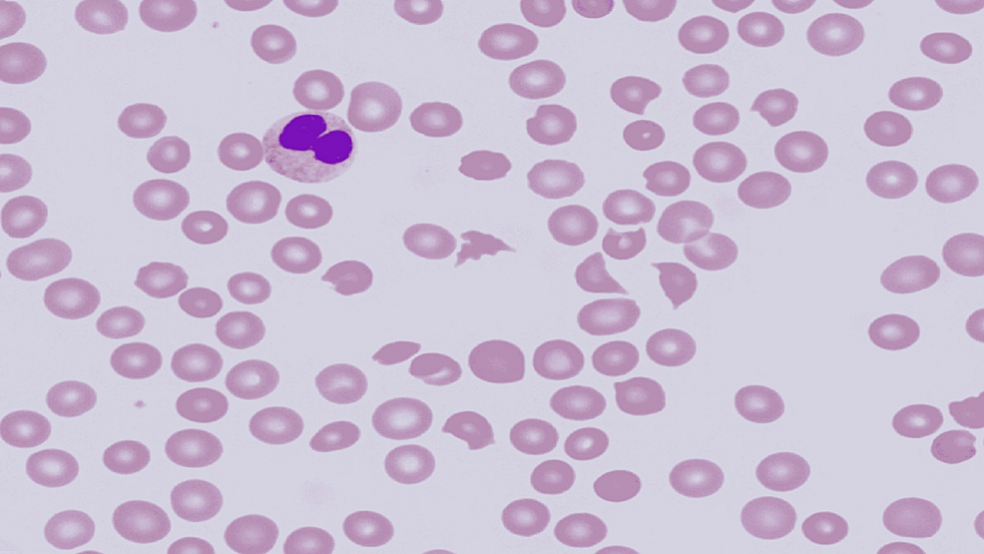
Peripheral blood smear revealing normocytic normochromic anemia, marked thrombocytopenia, mild to moderate anisopoikilocytosis, and schistocytes Image at 200x magnification.

A 76-year-old African American male with a medical background of diabetes mellitus type II and chronic hepatitis C presented to the emergency department (ED) with complaints of fatigue, nausea, significantly decreased urine output, penile pain, and scrotal swelling for 12-hours. Two days prior to his presentation to the ED, the patient had a penile implant procedure done, after which a Foley catheter was placed. On physical examination, he was mentally altered, fatigued, and had scrotal tenderness and swelling. Vitals on presentation were temperature of 98.4° F, blood pressure of 101/74 mmHg, heart rate of 98 beats/minute, and respiratory rate of 16 breaths/minute. Initial laboratory findings were significant for creatinine (Cr) of 4.7 mg/dL (baseline Cr 0.9 mg/dL), blood urea nitrogen (BUN) of 65 mg/dL, estimated glomerular filtration rate (eGFR) of 13 mL/min/1.73m^2^, potassium (K) of 6.3 mmol/L, platelet (PLT) count of 8,000/uL, hemoglobin (Hgb) level of 9.1 g/dL, white blood cell count (WBC) of 14,000/uL, D-dimer level was 3,228 ng/mL (normal: <243 ng/mL). Fibrinogen level, prothrombin time/international normalized ratio (INR), and partial thromboplastin time were within normal limits. Further hemolytic workup showed reticulocyte percentage of 3.60%, lactate dehydrogenase of 2,672 U/L (normal: 313-618 U/L), total bilirubin level of 3.1 mg/dL (normal: 0.1-1.3 mg/dL). A peripheral blood smear revealed normocytic normochromic anemia, marked thrombocytopenia, mild to moderate anisopoikilocytosis, and 8-10% schistocytes suggesting TTP (Figure 1).

The patient was admitted to the intensive care unit and was evaluated by the nephrology and hematology teams with a working diagnosis of TTP versus hemolytic uremic syndrome (HUS), complicated with AKI. Initially, hemodialysis was planned. However, after receiving one session of plasmapheresis, his urinary output and mental status improved. Subsequently, dialysis was held, and hyperkalemia was treated medically. Additionally, a disintegrin and metalloproteinase with a thrombospondin type 1 motif, member 13 (ADAMTS13) activity assay was sent on admission. It came back on the eighth day of admission with an activity level of <5%, confirming the diagnosis of TTP. The ADAMTS13 inhibitor screen was also positive, further confirming an acquired TTP.

The course of hospitalization was marked by an initial clinical improvement. Platelet count, lactate dehydrogenase (LDH), hemoglobin, Cr, and eGFR all improved after plasmapheresis was initiated (see Table 1). However, on the eighth day of admission, after the initiation of rituximab, plasmapheresis was held for one day to avoid rituximab washout. This led to a relapse of TTP, with platelet count dropping to 7,000 on the ninth day (Table 1). There was also a concomitant worsening of Cr (2.2) and eGFR (38). The patient was resumed on daily plasmapheresis, weekly rituximab, and also initiated on caplacizumab. Ultimately, there was a significant clinical improvement. Platelet count, hemoglobin, LDH, and Cr were all normalized (Table 1). ADAMTS13 level increased to >60, and plasmapheresis was stopped. The patient was subsequently discharged home with outpatient follow-up.

**Table 1:** Evolution of platelet count, hemoglobin level, lactate dehydrogenase level, creatinine level, eGFR, and ADAMTS13 activity over the course of hospitalization ADAMTS13 - a disintegrin and metalloproteinase with a thrombospondin type 1 motif, member 13; eGFR - estimated glomerular filtration rate

Day of admission	On admission	Day 2	Day 6	Day 9	Day 16	Day 21	Day 31
Platelet count (platelets/mcL)	8,000	50,000	102,000	7,000	120,000	93,000	181,000
Hemoglobin (g/dL)	7.3	8.4	9.7	8.7	7.6	12.7	9.9
Lactate dehydrogenase (U/L)	2672	742	607	4258	422	459	600
Creatinine level (mg/dL)	4.7	3.0	1.7	2.2	1.1	0.9	0.9
eGFR (mL/min/1.73m^2^)	13	26	45	38	>60	>60	>60
ADAMTS13 activity (%)	<5				67		

## Discussion

Thrombotic microangiopathy (TMA) is a group of fulminant clinical disorders characterized by thrombocytopenia, microangiopathic hemolytic anemia, and organ injury [1,2]. TMA can have a wide range of organ involvement; the kidney is one of the most affected. This is most likely due to the susceptibility of the glomerular circulation to microvascular occlusion and endothelial damage [4]. HUS and TTP are the two main forms of TMA, and their diagnosis was revolutionized with the advent of molecular testing. Historically, the distinction between these two disorders was mainly clinical: TTP has more neurologic involvement and worse thrombocytopenia, while HUS has more severe renal involvement [5]. Nowadays, TTP is diagnosed with low ADAMTS13 activity, typically less than 10% [4].

Indeed, TTP is mediated by ADAMTS13 deficiency, which can be inherited or acquired. ADAMTS13 is a metalloproteinase that cleaves von Willebrand factor (vWf) multimers. Low ADAMTS13 activity results in large vWf multimers and consequently increased platelet aggregation and microvascular occlusion [3].

Although renal involvement with proteinuria and hematuria are commonly observed in TTP, the consensus is that in TMAs, AKI is the hallmark of HUS. In a larger cohort of 75 TTP patients included in the Oklahoma registry, 58% had AKI, but only 10% were stage 3, and 4% required hemodialysis, concluding that severe AKI was uncommon among these patients [6].

Other authors disagree and consider that AKI is largely underestimated in TTP patients. In a retrospective study of 92 patients with TTP, Zafrany et al. identified 58.7% of patients with AKI, including 45.3% with stage 3 AKI. The renal replacement was needed for 25.9%. As per these authors, before the availability of ADAMTS13 activity measurements, renal involvement was used to distinguish TTP from HUS, which led to significantly underreported cases of TTP with severe AKI [5]. Although this is still a matter of debate, our case illustrates that severe AKI can still be one of the manifestations of true TTP. We wanted to emphasize that its presence should not be a criterion to rule out the diagnosis of TTP, possibly causing a delay in treatment.

Different pathophysiologic mechanisms could be at the origin of the kidney involvement in TTP. The thrombotic microangiopathy itself, with the unusually large number of vWf and platelet microthrombi in renal capillaries and arterioles, is thought to be the most likely mechanism. Indeed, renal microthrombi were identified in autopsy studies. Other potential causes include acute tubular necrosis due to hemodynamic instability, direct tubular or glomerular damage by the hemolysis products, drug-induced nephrotoxicity, and glomerulopathy [7].

## Conclusions

Our case illustrates that severe renal involvement such as stage 3 AKI can be a part of the clinical picture of TTP. Its presence should not be relied on to rule out the diagnosis. TTP is a fulminant disorder that has high mortality rates in the absence of specific treatment. The early recognition and the prompt initiation of plasmapheresis remain crucial in the management of these patients, possibly leading to complete recovery.
